# Supplementary material for: Genotype-Phenotype Correlations in Neurofibromatosis Type 1: Identification of Novel and Recurrent NF1 Gene Variants and Correlations with Neurocognitive Phenotype
Source: Genes (Basel). 2022 Jun 23;13(7):1130. doi: 10.3390/genes13071130 (PMC9316015; doi:10.3390/genes13071130)
Supplement: Supplementary file 1 [file genes-13-01130-s001.zip › genes-1726113-supplementary.pdf]

SUPPLEMENTARY TABLES

ID	Sex	Age	Clinical Features	Clinical Group
NF_2	F	34	- CALMs<br>- Cutaneous neurofibromas<br>- Axillary freckling<br>- Lisch nodules	G1
NF_54	F	47	- CALMs<br>- Cutaneous and subcutaneous neurofibromas<br>- UBOs	G1
NF_68	M	35	- CALMs<br>- Cutaneous and subcutaneous neurofibromas<br>- Spinal neurofibromas	G1
NF_83	F	70	- CALMs<br>- Cutaneous neurofibromas	G1
NF_89	F	56	- CALMs<br>- Subcutaneous fibromas	G1
NF_1	M	51	- CALMs<br>- Cutaneous neurofibromas<br>- Axillary and inguinal freckling<br>- Spine deformities (scoliosis)<br>- Learning disabilities<br>- Anxiety disorder	G2
NF_3	M	65	- Cutaneous neurofibromas<br>- Axillary and inguinal freckling<br>- Cardiovascular disorder (hypertension)<br>- Obesity<br>- Hepatic steatosis<br>- Spine (scoliosis) and bone deformities (pes cavus)	G2

NF_4	F	35	- CALMs<br>- Cutaneous neurofibromas<br>- Axillary freckling<br>- Spine deformities (scoliosis)<br>- Bone deformities (bilateral pes cavus)	G2
NF_5	F	44	- CALMs<br>- Subcutaneous neurofibromas<br>- Axillary freckling<br>- Spine deformities (scoliosis)<br>- Anxiety disorder<br>- Cardiovascular disorders (hypertension)	G2
NF_8	M	33	- Spinal neurofibromas<br>- Spine deformities (scoliosis)<br>- Learning disabilities<br>- Obesity	G2
NF_10	F	26	- CALMs<br>- Subcutaneous neurofibromas<br>- Spine (scoliosis) and bone (dural ectasia) deformities<br>- Cardiovascular disorders (hypertension)<br>- Learning disabilities	G2
NF_11	F	59	- CALMs<br>- Cutaneous and subcutaneous neurofibromas<br>- Spine (joint spondylosis) and bone (osteoporosis) deformities<br>- Anxiety disorder<br>- Sleep disorder<br>- Cardiovascular disorders (mitral and aortic insufficiency)	G2
NF_13	F	33	- CALMs<br>- Cutaneous neurofibromas<br>- Axillary freckling<br>- Lisch nodules<br>- UBOs<br>- Spine deformities (scoliosis)<br>- Obesity	G2
NF_20	M	18	- Cutaneous neurofibromas<br>- Inguinal freckling<br>- Lisch nodules<br>- Spine (kyphoscoliosis) and bone (foot deformities) deformities<br>- Learning disabilities	G2
NF_21	F	28	- CALMs<br>- Subcutaneous neurofibromas<br>- Spine (scoliosis, hydromyelia) and bone (short stature) deformities	G2

			- Cardiovascular disorders (mitral insufficiency)<br>- Plexiform neurofibroma in the right medial malleolus	
NF_25	F	26	- CALMs<br>- Cutaneous and subcutaneous neurofibromas<br>- Axillary and inguinal freckling<br>- Bone deformities (short stature)<br>- Hearing loss<br>- Learning disabilities	G2
NF_28	F	41	- CALMs<br>- Cutaneous neurofibromas<br>- Spine deformities (lost of the normal cervical lordosis)<br>- Obesity<br>- 25-OH Vitamin D deficiency	G2
NF_30	F	45	- CALMs<br>- Cutaneous and subcutaneous neurofibromas<br>- Spinal neurofibromas<br>- Axillary and inguinal freckling<br>- Spine and bone (short stature) deformities<br>- Cardiovascular disorder (hypertension)<br>- UBOs	G2
NF_38	M	27	- CALMs<br>- Behavior disorders<br>- Spine (scoliosis) and bone (short stature) deformities	G2
NF_40	F	41	- CALMs<br>- Cutaneous and subcutaneous neurofibromas<br>- Lisch nodules<br>- Vitamin D 25-OH deficiency<br>- Microcytic anemia<br>- Hearing loss<br>- Spine deformities (kyphoscoliosis)<br>- Fibrocystic mastopathy<br>- Learning disabilities	G2
NF_43	F	30	- CALMs<br>- Subcutaneous neurofibromas<br>- Spine deformities (scoliosis)<br>- Cardiovascular disorder (hypertension)<br>- Anxiety disorder<br>- Breast lump<br>- UBOs	G2
NF_45	M	70	- CALMs<br>- Subcutaneous neurofibromas	G2

			- Epilepsy syndrome<br>- Hypercholesterolemia<br>- Hearing loss<br>- Cardiovascular disorders (hypertension)	
NF_46	M	39	- CALMs<br>- Cutaneous and subcutaneous neurofibromas<br>- Spinal neurofibromas<br>- Plexiform neurofibromas of the right sheath<br>- Spine deformities (scoliosis)	G2
NF_47	M	36	- CALMs<br>- Subcutaneous neurofibromas<br>- Inguinal freckling<br>- Drug resistant epilepsy syndrome<br>- Spine deformities (scoliosis)	G2
NF_48	F	39	- CALMs<br>- Subcutaneous neurofibromas<br>- Axillary freckling<br>- UBOs	G2
NF_50	M	58	- CALMs<br>- Cutaneous neurofibromas<br>- Axillary and inguinal freckling<br>- Spine deformities (scoliosis)	G2
NF_51	F	29	- CALMs<br>- Cutaneous and subcutaneous neurofibromas<br>- Spinal neurofibromas<br>- Plexiform neurofibroma<br>- UBOs<br>- Erythrocythemia<br>- Spine (disc protrusions) and bone (short stature) deformities<br>- 25-OH Vitamin D deficiency<br>- Anxiety disorder	G2
NF_53	F	23	- CALMs<br>- Spine deformities (scoliosis)<br>- Cardiovascular disorder (coarctation of the aorta)<br>- Anxiety disorder<br>- Learning disabilities	G2
NF_56	M	56	- CALMs<br>- Subcutaneous neurofibromas<br>- Spine (scoliosis) and bone deformities<br>- Cardiovascular disorder (hypertension)<br>- Gastric disorder	G2

			- Multiple hepatic cysts	
NF_57	M	37	- CALMs<br>- Cutaneous neurofibromas<br>- Spine (kyphoscoliosis) and bone (short stature) deformities	G2
NF_59	M	52	- CALMs<br>- Cutaneous and subcutaneous neurofibromas<br>- Inguinal freckling<br>- Lisch nodules<br>- Spine (scoliosis) and bone (pectus carinatum) deformities<br>- Cardiovascular disorders (hypertension)	G2
NF_61	F	59	- Spine (scoliosis) and bone (dural ectasia) deformities<br>- Anxiety and depressive disorder	G2
NF_62	F	32	- CALMs<br>- Upper limb plexiform neurofibroma<br>- Spine deformities (scoliosis)<br>- Anxiety disorder<br>- D25-OH Vitamin deficiency	G2
NF_63	F	54	- CALMs<br>- Cutaneous neurofibromas<br>- Axillary and inguinal freckling<br>- Lisch nodules<br>- Spine deformities (scoliosis)<br>- Hypercholestrerolemia	G2
NF_66	M	63	- CALMs<br>- Subcutaneous neurofibromas<br>- Spinal neurofibromas<br>- Spine deformities (joint spondylosis)<br>- Cardiovascular disorders (hypertention)	G2
NF_71	F	40	- CALMs<br>- Cutaneous and subcutaneous neurofibromas<br>- Plexiform neurofibroma of the buttock region<br>- Spine deformities (scoliosis)<br>- Anxiety disorder<br>- 25-OH Vitamin D deficiency	G2

NF_77	M	20	- CALMs<br>- Cutaneous neurofibromas<br>- Axillary and inguinal freckling<br>- Spine (scoliosis) and bone (dural ectasia) deformities<br>- Cardiovascular disorders (mild tricuspid valve insufficiency)	G2
NF_80	M	32	- Cutaneous neurofibromas<br>- Lisch nodules<br>- UBOs	G2
NF_86	F	57	- CALMs<br>- Spine (scoliosis) and bone (osteoporosis) deformities	G2
NF_90	F	26	- CALMs<br>- Cutaneous and subcutaneous neurofibromas<br>- Spine (scoliosis) and bone deformities (foot deformities)<br>- UBOs	G2
NF_94	M	59	- CALMs<br>- Ischemic cerebrovascular disease<br>- Bone deformities (short stature)<br>- Mental retardation<br>- Glaucoma	G2
NF_96	F	70	- CALMs<br>- Cutaneous and subcutaneous neurofibromas<br>- Spine (scoliosis and joint spondylosis) and bone deformities (osteopenia and osteoporosis)<br>- Cardiovascular disorder (hypertension)<br>- 25-OH Vitamin D deficiency	G2
NF_100	F	57	- CALMs<br>- Cutaneous neurofibromas<br>- Spine deformities (joint spondylosis)<br>- Cardiovascular disorders (hypertension)<br>- Anxiety and depressive disorders<br>- Hepatic steatosis	G2
NF_102	M	33	- CALMs<br>- Cutaneous and subcutaneous neurofibromas<br>- Spine (kyphosis) and bone deformities<br>- UBOs	G2
NF_103	M	24	- CALMs<br>- Spine deformities (scoliosis)<br>- Freckling<br>- Lisch nodules	G2

			- Neurofibromas<br>- Thorax abnormalities	
NF_18	F	22	- Lisch nodules<br>- Spine (scoliosis) and bone deformities (non-ossifying fibroma of tibia)<br>- UBOs<br>- Learning disabilities<br>- Lumbar spinal MPNST	G3
NF_52	M	46	- CALMs<br>- Cutaneous and subcutaneous neurofibromas<br>- MPNST of the left limb<br>- GIST	G3
NF_127	M	19	- Intracranial MPNST	G3
NF_6	F	33	- CALMs<br>- Cutaneous and subcutaneous neurofibromas<br>- Inguinal freckling<br>- Spine (scoliosis) and bone (osteoporosis) deformities<br>- Cardiovascular disorders (heart murmur)<br>- Optic glioma	G4
NF_17	F	26	- CALMs<br>- Cutaneous neurofibromas<br>- Axillary and inguinal freckling<br>- Bone deformities (short stature)<br>- Spinal optic neurofibroma<br>- Malignant tumor at sacral region	G4
NF_26	F	35	- CALMs<br>- Subcutaneous neurofibromas<br>- Spinal neurofibromas<br>- Lisch nodules<br>- Spine (scoliosis) and bone (dural ectasia) deformities<br>- Multiple intraforaminal and paravertebral plexiform neurofibromas<br>- UBOs<br>- Anxiety disorder<br>- Pilocytic astrocytoma	G4
NF_35	M	55	- Cutaneous neurofibromas<br>- Orbito-temporal plexiform neurofibroma<br>- Chondropathy<br>- Diverticulosis<br>- 25-OH Vitamin D deficiency<br>- Spinal neurinoma	G4
NF_36	M	42	- CALMs<br>- Cutaneous and subcutaneous neurofibromas	G4

			- Axillary and inguinal freckling<br>- Spine deformities (scoliosis)<br>- Spinal neuromas	
NF_37	M	23	- CALMs<br>- Cutaneous and subcutaneous neurofibromas<br>- Spinal neurofibromas<br>- Spine deformities (scoliosis)<br>- Bilateral thalamic UBOs<br>- C2 ganglioneuroma	G4
NF_41	F	58	- CALMs<br>- Cutaneous neurofibromas<br>- Axillary and inguinal freckling<br>- Spine deformities (scoliosis)<br>- Hearing loss<br>- Cardiovascular disorders (hypertension)<br>- Optic glyoma<br>- MGUS	G4
NF_60	F	42	- CALMs<br>- Subcutaneous neurofibromas<br>- Spine (scoliosis) and bone (short stature) deformities<br>- UBOs<br>- 25-OH Vitamin D deficiency<br>- Astrocytoma	G4
NF_65	F	30	- CALMs<br>- Cutaneous and subcutaneous neurofibromas<br>- Spine deformities (scoliosis)<br>- Hearing loss<br>- Learning disabilities<br>- Cardiovascular disorder (hypertension)<br>- Optic glioma	G4
NF_67	M	52	- CALMs<br>- Cutaneous and subcutaneous neurofibromas<br>- Spinal neurofibromas<br>- Severe spastic tetraparesis<br>- Spinal astrocytoma	G4
NF_70	F	49	- Cutaneous and subcutaneous neurofibromas<br>- Spine deformities (scoliosis)<br>- Anxiety disorder<br>- Cardiovascular disorder (heart murmur)<br>- Microcytic anemia	G4

			- Vitamin D 25-OH deficiency<br>- Optic glioma	
NF_72	M	38	- Cutaneous neurofibromas<br>- Lisch nodules<br>- Spinal neurofibromas<br>- Spine deformities (scoliosis)<br>- Vitamin D 25-OH deficiency<br>- Optic glioma<br>- Astrocytoma	G4
NF_76	M	33	- CALMs<br>- Cutaneous neurofibromas<br>- Axillary freckling<br>- Lisch nodules<br>- Anxiety disorder<br>- Optic glioma	G4
NF_81	F	30	- CALMs<br>- Spinal neurofibromas<br>- Lisch nodules<br>- Spine deformities (scoliosis)<br>- Anxiety and depressive disorder<br>- Epilepsy syndrome<br>- Hearing loss<br>- Optic glioma<br>- Gangliocytoma<br>- Learning disabilities<br>- UBOs	G4
NF_82	F	30	- CALMs<br>- Subcutaneous neurofibromas<br>- Spinal neurofibromas<br>- Spine deformities (scoliosis)<br>- 25-OH Vitamin D deficiency<br>- UBOs<br>- Retroperitoneal ganglioneuroma	G4
NF_85	F	24	- Plexiform neurofibroma along the third branches of the trigeminal nerve<br>- Spine deformities (kyphoscoliosis)<br>- Optic glioma	G4
NF_99	F	47	- CALMs<br>- Cutaneous neurofibromas<br>- Anxiety disorder<br>- Spine deformities (scoliosis)<br>- Retroperitoneal ganglioneuroma	G4

NF_115	F	28	- CALMs<br>- Cutaneous and subcutaneous neurofibromas<br>- Lisch nodules<br>- Bone deformities (foot deformities)<br>- Optic glioma	G4
NF_15	M	31	- CALMs<br>- Cutaneous neurofibromas<br>- Spine deformities (loss of the normal cervical lordosis)<br>- Cardiovascular disorder (hypertension)<br>- Retinopathy<br>- Infundibular/hypothalamus lipoma	G5
NF_19	F	25	- CALMs<br>- Cutaneous and subcutaneous neurofibromas<br>- Axillary freckling<br>- Spine deformities (loss of the normal cervical lordosis)<br>- Depressive disorder<br>- Pheochromocytoma/paraganglioma	G5
NF_22	F	46	- CALMs<br>- Cutaneous and subcutaneous neurofibromas<br>- Lisch nodules<br>- Spine (scoliosis) and bone (osteoporosis) deformities<br>- Cardiovascular disorders (hypertension)<br>- Tethered Cord syndrome<br>- Spinal dysraphism<br>- Breast cancer	G5
NF_23	F	57	- CALMs<br>- Cutaneous neurofibromas<br>- Bone deformities (short stature)<br>- Breast cancer	G5
NF_29	M	52	- Spinal neurofibromas<br>- Lisch nodules<br>- Bone deformities (foot deformities)<br>- Epilepsy syndrome<br>- Seminoma	G5
NF_31	M	21	- CALMs<br>- Spinal neurofibromas<br>- Bilateral paravertebral plexiform neurofibroma<br>- Spine deformities (scoliosis)<br>- Brugada syndrome<br>- Learning disabilities	G5

			- Gallbladder adenomyomatosis	
NF_32	M	21	- CALMs<br>- Spinal neurofibromas<br>- Spine deformities (scoliosis)<br>- Brugada syndrome<br>- Adenomyomatosis	G5
NF_39	M	27	- Spine deformities (loss of the normal cervical lordosis)<br>- Anxiety disorder<br>- Vascular anomalies<br>- Femur ossifying fibromas	G5
NF_42	F	44	- CALMs<br>- Axillary and inguinal freckling<br>- Spine (joint spondylosis) and bone (cervical spondylosis) deformities<br>- Anxiety and depressive disorder<br>- Sleep disorder<br>- Nabothian cysts<br>- Fibromioma	G5
NF_55	F	52	- CALMs<br>- Cutaneous and subcutaneous neurofibromas<br>- Spine (scoliosis) and bone (osteopenia and foot deformities) deformities<br>- Hypercholesterolemia<br>- 25-OH Vitamin D deficiency<br>- Uterine myomas	G5
NF_73	M	37	- CALMs<br>- Cutaneous and subcutaneous neurofibromas<br>- Spinal neurofibroma<br>- Lisch nodules<br>- Spine (lost of the normal cervical lordosis) and bone (heterometry of the lower limbs) deformities<br>- Learning disabilities<br>- Pheochromocytoma	G5
NF_78	F	47	- CALMs<br>- Cutaneous neurofibromas<br>- Spine deformities (scoliosis)<br>- Hearing loss<br>- Uterine myoma<br>- Cholesteatoma (left ear)	G5

NF_79	F	45	- Cutaneous neurofibromas<br>- Lisch nodules<br>- Spine deformities (scoliosis)<br>- Dorso-lumbar CSF cyst<br>- Tubular adenoma	G5
NF_87	M	51	- Cutaneous neurofibromas<br>- Cardiovascular disorder (hypertension)<br>- Hypercholesterolemia<br>- Prostate cancer	G5
NF_92	M	56	- CALMs<br>- Spinal neurofibromas<br>- Malar facial plexiform neurofibroma<br>- Spine deformities (lost of the normal cervical lordosis)<br>- Cardiovascular disorders (mitral and aortic insufficiency)<br>- Hypercholesterolemia<br>- Lymphoma<br>- GIST	G5
NF_97	F	39	- CALMs<br>- Cutaneous and subcutaneous neurofibromas<br>- Spine (scoliosis) and bone deformities (pes cavus)<br>- Breast cancer	G5
NF_98	M	60	- CALMs<br>- Cutaneous and subcutaneous neurofibromas<br>- Spine deformities (joint spondylosis)<br>- Pheochromocytoma	G5
NF_101	M	21	- Cutaneous neurofibromas<br>- Spine (scoliosis) and bone (non-ossifying fibromas of tibia) deformities<br>- Cardiovascular disorders (aortic stenosis)<br>- Moyamoya syndrome	G5
NF_105	M	40	- CALMs<br>- Cardiovascular disorder (hypertension)<br>- Spinal neurofibromas<br>- Bladder heteroplasia<br>- Left thigh and leg malignant plexiform neurofibromas	G5

Table S1. Demographics and phenotypic features according to clinical groups (G1-G5) in the NF1 cohort. In column ID all NF1 patients were indicated with specific code (NF_ followed by an Arabic number) corresponding to the 'unique patient identifier' of the clinical centre. Age is referred to age at diagnosis. Group 1 (G1) includes patients with classical NF1 phenotype, including 6 or more café-au-lait spots (CALMs), axillary and inguinal freckling, two or more Lisch nodules and neurofibromas, without other manifestations of extra-cutaneous/ocular involvement; Group 2 (G2) encloses NF1 patients presenting the phenotypic features of G1 plus involvement of skeletal apparatus, central nervous system (epilepsy, intracranial vascular malformations, hamartomas/UBOs) and mental system (intellectual disability, anxiety/depression/sleep disorders), vascular system and anomalies of internal organs; Group 3 (G3) includes NF1 patients presenting the phenotypic features of G1 plus multi-apparatus involvement and histological diagnosis of MPNST; Group 4 (G4) includes NF1 patients presenting the phenotypic features of group G1 plus multi-apparatus involvement and neoplasms of the central and peripheral nervous systems; Group 5 (G5) includes NF1 patients presenting the clinical traits of G1 plus multi-apparatus involvement and neoplasms of variable grade of various organs and apparatus. UBOs: Unidentified bright objects; MGUS: Monoclonal Gammopathy of Undetermined Significance; MPNST: Malignant Peripheral Nerve Sheath Tumor, GIST: Gastrointestinal stromal tumor. F: female, M: male.

					NGS			
**ID**	***NF1* genetic mutation**	**Technique**			**Depth of the identified variants (TOT:WT:MUT)**	**Average depth**	**Depth minimum**	**Depth maximum**
		**NGS**	**RT-PCR/Sanger sequencing**	**MLPA**				
**NF_1**	c.(586+1_587-1)_(730+1_731-1)del	✓				125,15	15	297
**NF_2**	c.3G>A, p.?	✓			11:3:8	146,39	10	413
**NF_3**	c.3G>A, p.?		✓					
**NF_4**	c.3G>A, p.?		✓					
**NF_5**	c.1595T>G, p.Leu532Arg		✓					
**NF_6**	c.479+5G>A , p.Leu94fs	✓			91:47:44	132,61	15	340
**NF_8**	c.7884_7885del, p.(Phe2629Serfs*9)	✓			79:33:46	106,34	20	299
**NF_10**	c.3826C>T, p.Arg1276*		✓					
**NF_11**	c.3826C>T, p.Arg1276*		✓					
**NF_13**	c.6892_6897del, p.Ala2298_Val2299del	✓			249:142:107	217,75	13	651
**NF_15**	c.2619dup, p.Lys874*	✓			42:20:22	144,69	22	394
**NF_17**	c.6364+4A>G , p.Val2029Lysfs*7		✓					
**NF_18**	c.3326T>G, p.Leu1109*		✓					
**NF_19**	c.3826C>T , p.Arg1276*		✓					
**NF_20**	c.1783_1784del, p.Glu595fs		✓					
**NF_21**	c.(?_-383)_(*3522_?)del (whole NF1 deletion)	✓				99.5	15	185
**NF_22**	c.4982_4983del, p.Cys1661*	✓			123:68:55	129,83	26	373
**NF_23**	c.3496+1G>A , p.Tyr1106Leufs*28		✓					
**NF_25**	c.3496+1G>A, p.Tyr1106Leufs*28		✓					
**NF_26**	c.1246C>T, p.R416*		✓					

**NF_28**	c.1A>G, p.?	✓			13:1:12	132,34	15	382
**NF_29**	c.1595T>G, p.Leu532Arg		✓					
**NF_30**	c.3728T>C, p.Leu1243Pro	✓			252:135:117	121,39	17	343
**NF_31**	c.7686delG, p.Ile2563Phefs*40		✓					
**NF_32**	c.7686delG, p.Ile2563Phefs*40		✓					
**NF_35**	c.4269+2T>C, p.?		✓					
**NF_36**	c.6335T>C, p.Leu2112Pro		✓					
**NF_37**	c.(?_-383)_(*3522_?)del (whole NF1 deletion)	✓				96,21	12	175
**NF_38**	c.4768C>T, p.Arg1590Trp		✓					
**NF_39**	c.1009G>T, p.Glu337*	✓			261:130:131	172,53	22	495
**NF_40**	c.1466A>G, p.Tyr489Cys	✓			60:25:35	185,38	45	498
**NF_41**	c.1499_1501delinsAAA, p.Ile500_His501delinsLysAsn		✓					
**NF_42**	c.3502-3519 del, p.G1169-L1173del		✓					
**NF_43**	c.2352G>C, p.Trp784Cys	✓			26:11:15	122,02	52	352
**NF_45**	c.2307dup, p.Thr770HisfsTer6	✓			254:132:122	152,66	10	401
**NF_46**	c.2307dup, p.Thr770HisfsTer6	✓			254:132:122	150,09	11	419
**NF_47**	c.2307dup, p.Thr770HisfsTer6	✓			190:106:84	149,78	17	422
**NF_48**	c.1A>G, p.?	✓			23:9:14	142,97	24	391
**NF_50**	c.3497_3974del		✓					
**NF_51**	c.4381dup, p.Ile1461Asnfs*4	✓			63:35:28	124,44	25	335
**NF_52**	c.4309G>T, p.(Glu1437*)	✓			60:26:34	122,34	12	442
**NF_53**	c.1378dup, p.Ile460Asnfs*10	✓			212:108:104	156,18	11	426
**NF_54**	c.1639G>T, p.Glu547*	✓			81:38:43	162,43	10	442
**NF_55**	c.4537C>T, p.Arg1513*	✓			49:23:26	181,98	33	495
**NF_56**	c.2409+1G>A, p.?; c.2375T>A, p.Leu792His	✓			33:17:16	158,46	32	445
**NF_57**	c.1381C>T, p.Arg461*	✓			167:85:82	134,68	21	374
**NF_59**	c.4270-2A>G, p.Ile1424_Gln1426del	✓			46:28:18	144,01	20	402

**NF_60**	c.1756_1759del, p.Thr586Valfs*18	✓			35:16:19	125,90	19	339
**NF_61**	c.4367+2T>C , p.?		✓					
**NF_62**	c.1260+1G>A, p.Ser421fs	✓			101:58:43	172,98	17	488
**NF_63**	c.4733C>A, p.(Ser1578Tyr)	✓			159:79:80	194,49	16	509
**NF_65**	c.(?_-383)_(*3522_?)del (whole NF1 deletion)	✓				82,03	13	154
**NF_66**	c.2665 A>G, p.Thr889Ala	✓			54:35:19	168,88	15	415
**NF_67**	c.6084+1G>A , p.?	✓			199:105:94	224,34	26	542
**NF_68**	c. 6792C>A , p.Ala2253_Lys2286del		✓					
**NF_70**	c.2409+1G>C , p.?	✓			35:24:11	196,06	32	510
**NF_71**	c.2409+1G>C , p.?	✓			53:28:25	184,97	25	506
**NF_72**	c.7884_7885del, p.Phe2629Serfs*9	✓			242:126:116	237,30	11	592
**NF_73**	c.4923G>A, p.Trp1641*		✓					
**NF_76**	c.1845+1_1845+5del, p.Ala548_Lys615del	✓			92:47:45	194,93	15	529
**NF_77**	c.2326G>A, p.Ala776_Gln803del		✓					
**NF_78**	c.2329T>C, p.Trp777Arg	✓			37:23:14	226,59	14	592
**NF_79**	c.3916C>T, p.Arg1306*, c.1975C>T, p.Arg659Trp	✓			145:71:74	211,39	10	544
**NF_80**	c.4278G>C, p. Gln1426His	✓			58:30:28	151,59	15	385
**NF_81**	c.5819del, p.(Lys1940Serfs*18)	✓			131:61:70	175,05	19	459
**NF_82**	c.8051-1 G>C , p.?	✓			63:23:40	142,41	17	387
**NF_83**	c.5673T>G, p.Ser1891Arg	✓			111:60:51	221,46	13	587
**NF_85**	c.6621dup, p.Trp2208Valfs*13	✓			285:145:140	162,50	12	423
**NF_86**	c.4923G>A, p.Trp1641*	✓			156:78:78	181,44	11	431
**NF_87**	c.7259C>A, p.Ala2420Asp		✓					
**NF_89**	c.(4661+1_4662-1)_(7258+1_7259-1)dup	✓		✓				
**NF_90**	c.(4661+1_4662-1)_(7258+1_7259-1)dup	✓		✓				
**NF_92**	c.4278G>C. p.Gln1426His	✓			71:35:36	165,75	26	447
**NF_94**	c.2252-3T>G, p.?	✓			195:85:110	134,13	24	369

**NF_96**	c.(?_-383)_(*3522_?)del<br><br>(whole *NF1* deletion)	✓				79,50	12	165
**NF_97**	c.4515-2A>G , p.?	✓			28:5:23	151,35	22	395
**NF_98**	c.7089dup, p.Asn2364*		✓					
**NF_99**	c.2851G>T, p.Val951Phe		✓					
**NF_100**	c.4537C>T , p.Arg1513*	✓			41:50:11	142,88	21	394
**NF_101**	c.1329delT, p.Phe443Leufs*29		✓					
**NF_102**	c.1381C>T, p.Arg461*		✓					
**NF_103**	c.2251 G>C , p.Asp668Glufs*9		✓					
**NF_105**	c.C7532T, p.(Ala2532Val)		✓					
**NF_115**	c.2446C>T, p.Arg816*		✓					
**NF_127**	c.2540T>G, p.Leu847Arg		✓					

Table S2. Sequencing techniques performed and NGS details. Next-generation sequencing (NGS), Real Time PCR (RT-PCR), Sanger sequencing, and Multiple Ligation Probe Amplification (MLPA) were applied to *NF1* mutation analysis. NGS details including depth of the identified *NF1* variants, average depth, minimum and maximum depth are shown.
